# The emerging roles and mechanisms of exosomal non-coding RNAs in the mutual regulation between adipose tissue and other related tissues in obesity and metabolic diseases

**DOI:** 10.3389/fendo.2022.975334

**Published:** 2022-08-18

**Authors:** Shifeng Pan, Yongfang Chen, Jie Yan, Fei Li, Xinyu Chen, Xingyu Xu, Hua Xing

**Affiliations:** ^1^ College of Veterinary Medicine, Yangzhou University, Yangzhou, China; ^2^ Jiangsu Co-innovation Center for Prevention and Control of Important Animal Infectious Diseases and Zoonoses, Yangzhou University, Yangzhou, China; ^3^ Department of Animal Science, Washington State University, Pullman, WA, United States

**Keywords:** exosomes, non-coding RNAs, adipose tissue and other related tissues, interaction, cell-to-cell communication

## Abstract

Exosomes (EXs) are the major types of extracellular vesicles (EVs) of 30-100 nm diameter that can be secreted by most cells to the extracellular environment. EXs transport endogenous cargoes (proteins, lipids, RNAs, etc.) to target cells and thereby triggers the release of these bioactive components, which then play important roles in regulating numerous biological processes under both physiological and pathological conditions. Throughout the studies in recent years, growing evidences have shown that EXs-derived non-coding RNAs (EXs-ncRNAs) are emerging as key players in cell-to-cell communication between adipose tissue and other related tissues in obesity and metabolic diseases. In this review, we will summarize the recent findings about EXs-ncRNAs, especially focus on the following aspects: 1) the biogenesis of EXs and emerging roles of EXs-ncRNAs, 2) the role of EXs-ncRNAs (EXs-miRNAs, EXs-lncRNAs, EXs-circRNAs, etc.) that were secreted by adipose-related tissues in promoting the differentiation of preadipocytes into mature and fully functional adipocytes, and 3) the crosstalk between the adipose tissue derived EXs-ncRNAs and the development of insulin resistance, obesity and various cancers. This review aims to reveal the emerging roles and mechanisms of EXs-ncRNAs in the mutual regulation of adipose tissue and its related tissues in obesity and metabolic diseases, so as to provide references for elucidating the etiology of obesity and related metabolic diseases and screening novel therapeutic targets.

## Introduction

Mammalian adipose tissue can be divided into two major types, namely, white adipose tissue (WAT) and brown adipose tissue (BAT). It is currently believed that white and brown adipocytes arise from distinct embryonic precursors and their biological functions are totally different. According to classical view, WAT is mainly used as an energy storage organ to accumulate fat in the form of triglycerides (TG), and in times of caloric need, white adipocytes provide a long-term metabolic fuel *via* lipolysis and the release of fatty acids. However, adipose tissue is not only an important energy storage organ, it is also the largest endocrine organs in the body, which can be involved in functions such as hormone (a variety of adipokines, such as adiponectin and leptin, etc.) secretion (1) and immune function (2). Adipose tissue dysfunction is strongly associated with obesity and metabolic complications (3) by regulating the functions of various metabolic tissues and organs, and plays an important role in the pathogenesis of type 2 diabetes (T2DM) (4, 5), insulin resistance (IR) (6, 7) and cardiovascular diseases (8). Furthermore, other tissues and secreting organs in the body can also secrete specific cytokines (such as insulin, irisin, etc.), and in turn participate in regulating adipose tissue functions, indicating that adipose tissue and its related tissues and organs have closely-coupled interaction, which have drawn interest from researchers of quite different fields. However, so far, many key problems about the interaction, especially the mutual regulation mechanisms, have not been well clarified yet. Exosomes (EXs) are nanosized membranous vesicles secreted by a variety of cells, which can encapsulate and transfer a large number of non-coding RNAs (ncRNAs), proteins, lipids and numerous other functional compounds, and sever as important mediators of cell-to-cell communication. In recent years, growing evidences have strongly demonstrated that EXs-derived non-coding RNAs (EXs-ncRNAs) play a crucial role in signal transmission between adipose tissue and other related metabolic tissues (9, 10), and regulate the pathogenesis of obesity and related metabolic syndrome. In the body, adipose tissue can regulate its related tissues or organs by secreting EXs-ncRNAs (11–14), while EXs-ncRNAs from other related tissues can also affect lipid metabolism and fat deposition in adipose tissue. However, it is undoubted that ncRNAs are only one of many functional compounds that can be encapsulated and transferred by EXs, other functional components, such as proteins, are also important. In this review, we specifically focus on the effects of ncRNAs to summarize, mainly due to the following reasons, on one hand, great efforts have focused on understanding protein-coding RNAs and their involvement in the regulation of adipocyte physiology and subsequent role in obesity, while diverse findings have suggested that adipose tissue dysfunction in obesity might be also dependent on specific alterations of ncRNAs expression pattern. On the other hand, notably, ncRNAs (especially EXs-ncRNAs) are a hot research topic in recent years and have attracted particular attention (15, 16). However, so far, there are few systematic reviews focusing on the emerging roles and mechanisms of EXs-ncRNA in the mutual regulation between adipose tissue and related tissues in obesity and metabolic diseases. This review mainly summarizes the following aspects: 1) the biosynthesis of EXs and biological functions of EXs-ncRNAs, 2) he emerging roles and potential mechanisms of EXs-ncRNAs (EXs-miRNAs, EXs-lncRNAs and EXs-circRNAs) that were derived from adipose tissue related tissues in regulating terminal differentiation of preadipocytes into adipocytes, and 3) the interaction between the EXs-ncRNAs that were derived from adipose tissue and IR as well as the development of obesity and various cancers. The review aims to elucidate the signal transmission and the potential molecular mechanisms between adipose tissue and other related tissues in obesity and metabolic diseases, so as to provide theoretical support for the prevention and treatment of obesity and its related complications.

## Biogenesis of EXs and biological functions of EXs-ncRNAs

EXs are nanometer-sized vesicles with a diameter of 30-100 nm that are released to the outside of the cell during environmental stimulation or cell activation (17). EXs can be directly isolated from various body fluids (blood, urine, cerebrospinal fluid, semen, saliva, etc.) and the cell culture supernatant. EXs biosynthesis mainly consists of two successive stages: 1) double invagination at the plasma membrane, forming multiple intraluminal vesicles (ILVs) and resulting in the formation of multivesicular bodies (MVBs); 2) the release of MVBs, then ILVs are secreted into the extracellular environment as EXs (18, 19). Thus, in essence, EXs are the intermediate ILVs encapsulated within MVBs. When these polyvesicles are fused with the cell membrane, multiple luminal vesicles are secreted out of the cell. Once secreted, EXs can reach recipient cells and deliver their cargoes, thereby regulating the biological functions of the recipient cells. The cell-to-cell interactions mediated by EXs generally produced by three ways: 1) transmembrane proteins of donor cells derived EXs act directly with signal receptors on the membrane of the recipient cells (2); the membrane of donor cells EXs fuse with the membrane of the recipient cells, whose cargoes are then transferred directly into the cytoplasm of recipient cells; 3) the donor cells EXs are fully swallowed into the recipient cells, then the bioactive substances encapsulated were released.

Numerous studies have shown that EXs can package and transfer a variety of biological active molecules, including proteins (20, 21), lipid (22, 23), messenger RNA (mRNA) (24) and a large number of ncRNAs. These bioactive molecules with EXs are released into the various kinds of body fluids to participate in the regulation of a variety of pathological and physiological processes, such as neuron degeneration (25, 26), tumor (27, 28), diabetes (17), and so on. EXs carry surface-labeled proteins such as CD63, CD9, CD81, Alix, TSG101 and HSP70 on their surface, and the isolated EXs can be identified by detection of these specific proteins. At present, studies on EXs mainly focus on their classification, isolation methods and biological functions in the progression and treatment of diseases (18, 19, 29, 30). In addition, previous studies have also found that EXs secreted by other tissues of the body can participate in the regulation of adipocyte differentiation and fat metabolism by regulating the expression of adipogenic differentiation-specific genes, lipogenic enzymes, lipid-metabolizing enzymes and other transcription factors. Additionally, EXs can reflect the conditions of obesity and related metabolic diseases and are potential targets for diagnosis and treatment of obesity with important clinical value. NcRNAs refer to molecules that lack protein-coding regions, which have become a hot topic of increasing concern and can regulate >60% of human genes (31, 32) expression at transcriptional, post-transcriptional, and translational levels (33, 34). Since 2007, accumulating evidences have demonstrated that numerous ncRNAs can be encapsulated and transported by EXs, the most attractive of which are miRNAs, lncRNAs, and circRNAs, explaining their roles in intercellular communication (35, 36). Notably, EXs-ncRNAs exhibit diverse expression patterns in different cells or various physiological and pathological conditions, indicating the potential role of EXs-ncRNAs in occurrence and development of different diseases (37, 38). When the EXs-ncRNAs undergo tissue-specific changes due to diverse internal or external disorders, they can cause tissue dysfunction and diseases, indicating that EXs-ncRNAs are promising diagnostic and therapeutic tools for various human diseases (16, 39–41). A large number of studies dealing with circulating EXs and their cargoes have proved that EXs-miRNAs, EXs-lncRNAs and EXs-circRNAs are closely involved in human health and the initiation and development of various diseases (42). Therefore, we specifically focus on the mutual crosstalk between adipose tissue and other related tissues in obesity and metabolic diseases mediated by EXs-ncRNAs, which has become a research hotspot in recent years (43), is able to provide support for the diagnosis and treatment of obesity and related metabolic diseases.

## EXs-derived miRNAs regulate adipogenic differentiation

miRNAs are single-stranded non-coding RNAs with a length of 19-25 nt, which can participate in the regulation of a variety of biological processes by post-transcriptional inhibition of target gene expression mainly by promoting mRNA degradation and/or inhibiting protein translation. Numerous previous studies have demonstrated that miRNAs are significantly correlated with adipogenic differentiation of preadipocytes. In mouse 3T3-L1 preadipocytes, it was found that during the adipogenic differentiation, the expression of miR-17-92 was gradually increased, and miR-17-92 overexpression significantly induced adipogenic differentiation and increases TG accumulation (44). In addition, studies had demonstrated that miR-146b up-regulation can significantly reduce the glucose consumption in pig primary adipocytes and inhibit its adipogenic differentiation by suppressing the expression of insulin receptor substrate 1 (IRS1). On the contrary, the expression levels of GLUT4 and IRSI were significantly increased after miR-146b expression was inhibited, and the adipogenic differentiation was also obviously enhanced (45). Moreover, miR-34a was able to inhibit porcine preadipocyte differentiation and lipid accumulation by inhibiting PDGFRα expression (46). Similarly, studies in porcine intramuscular preadipocytes have shown that miR-17-5p overexpression can significantly inhibit the expression of NCOA3 and its related adipogenic marker genes PPAR-γ and FABP4, thus inhibited adipogenic differentiation and TG deposition (47). Previous studies in our group also demonstrated that miR-130b and miR-374b were able to significantly inhibit the subcutaneous fat deposition of weaned piglets under maternal low-protein levels by inhibiting the expression of PPAR-γ and C/EBP-β, respectively (48). Our *in vitro* studies also showed that both miR-130b and miR-374b overexpression were able to significantly inhibit the adipogenic differentiation of primary cultured porcine subcutaneous preadipocytes (49). Taken together, these above results suggest that miRNAs are closely associated with the regulation of adipogenic differentiation.

MiRNAs play key roles in adipogenic differentiation. However, miRNAs are unstable and easy to be degraded, which greatly limit their broad clinical application and development. However, EXs have a double-membrane structure, and EXs-miRNAs are well resistant to ubiquitous RNA enzymes, extreme temperatures and pH levels, and can be even preserved for a long time in the extracellular environment. And EXs-miRNAs can be swallowed by target cells by plasma membrane fusion and endocytosis, and exert functional effects in various signaling pathways just like endogenous miRNAs. These suggest that EXs-miRNAs play important roles in regulating the adipogenic differentiation of preadipocytes, after entering the circulation system, EXs can play a regulatory role in the target tissues through the specific proteins carried on EXs membrane and the bioactive substances in the membrane. Researches on mice have shown that no apparent immune rejection and inflammatory response were observed after repeated administration of a relatively low dose of mouse or human cell-derived EXs for a long time (41, 50, 51), suggesting low antigenicity and toxicity of EXs (52).

EXs contain a variety of bioactive substances, among which miRNAs play an important role in regulating cell growth, differentiation and metabolism. And the special structure of EXs protects the encapsulated miRNAs from RNase degradation in body fluids. These suggest that EXs may become the important carrier of miRNAs drugs in the future. Studies have shown that circulating miRNAs in animals are mainly derived from adipose-derived cells (53), and EXs-miRNAs secreted by other tissues can also be taken up, absorbed and utilized by adipose tissue, suggesting that EXs-miRNAs play an important role in regulating the interaction between adipose tissues and other related tissues. In physiological state, skeletal muscle-derived EXs can inhibit adipogenesis of porcine intramuscular preadipocytes (54). EXs-miR-130a-3p that was secreted by liver tissue can be taken up by 3T3-L1 preadipocytes, and then inhibited the adipogenic differentiation by inhibiting FASN and PPAR-γ expression (55). Furthermore, EXs-miR-155 derived from adipose tissue macrophages were able to inhibit adipogenic differentiation by inhibiting PPAR-γ expression, and regulated the insulin sensitivity both *in vivo* and *in vitro* (56). Compare with control cells, high levels of miR-122 were found only in adipose tissue-derived exosomes (EXs-AT) and EXs-AT-treated cells. Overexpression of miR-122 promoted adipogenesis, while miR-122 inhibition prevented adipogenesis by regulating VDR, SREBF1, PPAR-γ, LPL and adiponectin (57). Our previous results *in vitro* study also showed that overexpressed miR-130b could be successfully packaged into EXs, and EXs-miR-130b could be taken up into porcine subcutaneous preadipocytes and inhibited the adipogenic differentiation by inhibiting the expression of PPAR-γ and its related genes (49). In addition, our *in vivo* studies also showed that tail-vein injection of EXs-miR-130b for 10 d significantly alleviated the glucose tolerance of HFD induced obese mice, and also the body weight, epididymal fat content and epididymal fat weight/body weight ratio were all significantly reduced (58). These above findings revealed that EXs-miR-130b was able to exert a biological function of inhibiting fat deposition both *in vivo* and *in vitro* models.

In pathological conditions, EXs secreted by pancreatic cancer cells could induce lipolysis of mouse 3T3-L1 preadipocyte and human mature adipocytes through adrenal medulla hormone and its receptor (59). EXs of human hepatocellular carcinoma cell line HepG2 could be taken up by adipocytes and the activated both the phosphorylated kinases and NF-κB pathways in adipocytes, thereby promoting tumor growth by enhancing angiogenesis (60). In addition, EXs-miR-34a secreted by mature adipocytes was able to inhibit M2 polarization of macrophages by downregulating KLF4 (61). Moreover, EXs derived from M2 macrophages (M2D-EXs) were able to inhibit adipogenesis and promote osteogenesis of BMSCs through the miR-690/IRS-1/TAZ axis (62). Additionally, EXs-miR-144 in breast cancer cells significantly promoted beige/brown staining by down-regulating MAP3K8/ERK1/2/PPAR-γ axis, and EXs-miR-126 promoted adipose tissue remodeling by disrupting IRS-1/PI3K/GLUT4 signaling pathways, activating the AMPK/autophagy pathways and stabilizing the expression of HIF1α (63). In addition, EXs derived from lung cancer cells could be taken up and utilized by human adipose mesenchymal stem cells (hAMSCs) and inhibited the adipogenic differentiation by activating TGF-β signaling pathway (64). EXs-miR-92a-3p in serum of chronic myeloid leukemia inhibited adipogenic differentiation of adipocytes by decreasing C/EBP-α expression (65). Moreover, EXs derived from gastric cancer (GC) cells significantly suppressed adipogenesis in AMSCs as characterized by decreased lipid droplets. Overexpression of EXs-miR-155 secreted from GC cells suppressed adipogenesis and promoted brown adipose differentiation by targeting C/EBP-β, accompanied by downregulated C/EBP-α and PPAR-γ and upregulated UCP1. Also *in vivo* study, it was found that overexpression of GC cells secreted EXs-miR-155 improved CAC *in vivo*, which was characterized by fat loss, suppressed expression of C/EBP-β, C/EBP-α and PPAR-γ in AMSCs, and higher expression of UCP1 (66). All above results uniformly suggested that EXs-miRNAs could act as an intermediary mediating the interaction between adipose tissue and its related tissues/organs.

Although numerous studies have demonstrated that EXs-miRNAs are able to inhibit adipogenic differentiation, on the contrary, some other studies have confirmed that some EXs-miRNAs can significantly promote adipogenic differentiation. For example, differential miRNA profiles in EXs of rat adipose tissue and adipose-derived stem cells (ADSCs) have been analyzed in a previous study through high-throughput sequencing technologies, and the results showed that EXs of adipose tissues were enriched with 45 miRNAs compared with ADSCs, among which 14 miRNAs, such as miR-30a-5p, miR-148a-3p and miR-450a-5p, were involved in the regulation of adipose tissue formation. Further exploration showed that EXs-miR-450a-5p in adipose tissue promoted adipogenic differentiation of rat ADSCs through inhibiting expression of its target gene WISP2 (67). These above results suggest that EXs-miRNAs play a dual regulatory role in adipogenic differentiation of adipocytes (**Table 1**).

**Table 1:** EXs and/or miRNAs regulate adipogenic differentiation.

EXs and/or ncRNAs	Donor cells/organs	Recipient cells/organs	Functions	Mechanisms	References
MiR-17-92	–	3T3-L1 preadipocytes	Induces adipogenic differentiation and increases TG accumulation	Negatively regulates tumor-suppressor Rb2/p130	(44)
MiR-146b	–	Porcine primary preadipocytes	Inhibits adipogenic differentiation	Suppresses the expression of IRS1	(45)
MiR-34a	–	Porcine preadipocyte	Inhibits differentiation and lipid accumulation	Inhibits the expression of PDGFRα	(46)
MiR-17-5p	–	Porcine intramuscular preadipocytes	Inhibits adipogenic differentiation and TG deposition	Inhibits the expression of NCOA3, PPAR-γ and FABP4	(47)
MiR-130b and MiR-374b	–	Subcutaneous fat of weaned piglets	Inhibits the subcutaneous fat deposition	Inhibits the expression of PPAR-γ and C/EBP-β respectively	(48, 49)
EX-miR-130a-3p	Liver tissue	3T3-L1 preadipocytes	Inhibits the adipogenic differentiation	Inhibits FASN and PPAR-γ expression	(55)
EX-miR-155	Adipose tissue macrophages	Mice	Inhibits adipogenic differentiation	Inhibits PPAR-γ expression and regulates the insulin sensitivity	(56)
MiR-122	–	Mice	Inhibits miR-122 expression and prevents adipogenesis	Regulates VDR, SREBF1, PPAR-γ, LPL and adiponectin	(57)
EXs-miR-130b	–	Porcine subcutaneous preadipocytes	Inhibits the adipogenic differentiation	Inhibits the expression of PPAR-γ and its related genes	(49)
EXs	Pancreatic cancer cells	3T3-L1 preadipocyte and human mature adipocytes	Induces lipolysis	Regulates the expression of adrenal medulla hormone and its receptor	(59)
EXs	HepG2	Adipocytes	Enhances angiogenesis	Activates both the phosphorylated kinases and NF-κB pathways	(60)
EXs-miR-34a	Mature adipocytes	Macrophages	Inhibits M2 polarization of macrophages	Downregulates expression of KLF4	(61)
EXs	M2 macrophages	BMSCs	Inhibits adipogenesis and promotes osteogenesis of BMSCs	Increases the miR-690/IRS-1/TAZ axis	(62)
EXs-miR-144	Breast cancer cells	Adipocyte	Promotes beige/brown staining	Downregulates MAP3K8/ERK1/2/PPAR-γ axis	(63)
EXs-miR-126	–	Adipose tissue	Promotes adipose tissue remodeling	Disrupts IRS-1/PI3K/GLUT4 signaling pathways, activates the AMPK/autophagy pathways and stabilizes the expression of HIF1α	
EXs	Lung cancer cells	hAMSCs	Inhibits the adipogenic differentiation	Activates TGF-β signaling pathway	(64)
EXs-miR-92a-3p	Serum of CML	Adipocytes	Inhibits adipogenic differentiation	Decreases C/EBP-α expression	(65)
EXs-miR-155	GC cells	AMSCs	Suppresses adipogenesis	Downregulates C/EBP-α and PPAR-γ and upregulates UCP1	(66)
EXs-miR-450a-5p	Adipose tissue	ADSCs	Promotes adipogenic differentiation	Inhibits expression of WISP2	(67)

## EXs-derived circRNAs regulate adipogenic differentiation

CircRNAs are a fresh class of non-coding RNA molecules widely present in the cytoplasm of eukaryotic cells. CircRNAs form a covalently closed circular continuous loop by “reverse splicing” and are highly conserved among different species, and this kind of RNA has no 5’-end cap and 3’-end polyadenylic acid tail, which makes it resistant to RNA degradation and can be widely existed in various cells with high stability. A large number of previous studies have proved that circRNAs can be used as competitive endogenous RNAs (ceRNAs), especially as miRNAs inhibitors, to regulate the activity and transcription level of miRNAs target genes and participate in transcriptional or post-transcriptional gene expression regulation. Studies have also shown that circRNAs can participate in the regulation of adipose tissue growth, adipogenic differentiation and other processes, and play an important role in regulating body fat deposition. For example, compared with normal adipose tissue, circRNA has_circ_0075932 is significantly increased in adipose tissue of obese people (68). Studies have also shown that knock-down of circular RNA H19 promotes adipogenic differentiation of hADSCs and transfers SREBP1 from cytoplasm to nucleus *via* targeting of PTBP1 (69). In addition, ceRNA circ FUT10 can competitively inhibit the expression of its target gene let-7c and suppresses the adipogenic differentiation of Qinchuan bovine preadipocytes by upregulating the expression of PPAR-γ co-activator -1α (PGC1α) (70). In addition, through acting as a “sponge” of miR-138-5p, circ SAMD4A can increase the expression of EZH2 and promotes the differentiation of adipocytes, while the adipogenic differentiation is significantly inhibited after circ SAMD4A is knocked out (71). These results suggest that circRNAs play vital roles in the regulation of adipogenic differentiation under physiological states.

Studies in disease models have demonstrated that EXs-circRNAs in plasma have specific expression characteristics and can be used as biomarkers for numerous diseases screening. For example, the expression level of ciRS-133 in plasma EXs of GC patients is obviously increased, which significantly promotes white adipose browning (72). Studies *in vitro* have found that EXs derived from GC cells can deliver ciRS-133 to adipocytes and further activates PRDM16, which then promotes the differentiation of preadipocytes into brown-like cells by targeting miR-133 (72). Compared with healthy plasma EXs, plasma EXs derived from osteoporosis patients have significantly higher level of Hsa_circ_0006859, which can significantly suppress osteoblastic differentiation and promote adipogenic differentiation of human bone marrow-derived mesenchymal stem cells (hBMSCs) by upregulating miR-431-5p and thus inhibiting ROCK1 (73). These above results suggest that circRNAs in EXs and non-EXs can regulate adipogenic differentiation in both physiological and pathological processes (**Table 2**).

**Table 2:** EXs and/or circRNAs regulate adipogenic differentiation.

EXs and/or ncRNA	Donor cells/organs	Recipient cells/organs	Functions	Mechanisms	References
Circ RNA H19	–	hADCSs	Inhibits adipogenic differentiation	Transfers SREBP1 from cytoplasm to nucleus *via* targeting of PTBP1	(69)
Circ FUT10	–	Qinchuan bovine preadipocytes	Suppresses adipogenic differentiation	Inhibits the expression of let-7c and upregulate the expression of PGC1α	(70)
Circ SAMD4A	–	Adipocytes	Promotes adipogenic differentiation	Increases the expression of EZH2 through acting as a “sponge” of miR-138-5p	(71)
EXs-ciRS-133	Plasma EXs of GC patients	White adipose	Promotes white adipose browning	Activates PRDM16, and targets the expression of miR-133	(72)
	GC cells	Adipocytes	Promotes the differentiation of preadipocytes into brown-like cells		
Hsa_*circ*_*0006859*	Patients with osteoporosis	hBMSCs	Promotes adipogenic differentiation	Upregulates the expression of miR-431-5p and inhibits ROCK1	(73)

## EXs-derived lncRNAs regulate adipogenic differentiation

LncRNAs are a kind of RNA molecules with transcription length ≥200 nucleotides. Similar to miRNAs, lncRNAs do not have protein coding functions, but can regulate the expression of their target genes at epigenetic, transcriptional and post-transcriptional levels. At present, several studies have shown that lncRNAs are closely related to adipogenic differentiation. For example, a study in humans showed that lncRNA HOTAIR specifically expressed in gluteus muscle could significantly promote abdominal fat-derived preadipocyte differentiation by increasing the expression of PPAR-γ and LPL (74). What’s more, studies on ADSCs isolated from the inguinal adipose tissue of female rats have shown that lncRNA-ADI expression is significantly increased during adipocyte differentiation, while specific inhibition of lncRNA-ADI significantly reduces the adipogenic differentiation ability of ADSCs. These results indicate that lncRNA-ADI plays an important role in promoting adipogenic differentiation of ADSCs. Further research has shown that lncRNA-ADI significantly promotes adipogenic differentiation of ADSCs, mainly through inhibiting miR-449a expression, which then further increases the CDK6 translation and activates pRb-E2F1 pathway (75). *In vitro* experiments have shown that the expression of lncRNA HOXA11-AS1 is gradually increased with the differentiation process of hADSCs, while the expression of adipogenesis key genes C/EBP-α, DGAT2, CIDEC and perilipin are significantly inhibited, and their adipogenic differentiation ability and lipid accumulation are significantly reduced after specific knockdown of lncRNA HOXA11-AS1 in hADSCs. In addition, lncRNA HOXA11-AS1 expression is significantly increased in obese patients compared with the non-obese patients (76). Moreover, in hBMSCs, during the process of osteogenic differentiation, the expression of LOXL1-AS1 is gradually decreased, and overexpression of LOXL1-AS1 can promote the expression of Hmga2 protein by inhibiting the expression of miR-196a-5p, thus switching from adipogenic differentiation to osteogenic differentiation (77). Studies on chicken, lncRNAs such as XLOC_068731, XLOC_022661, XLOC_045161, XLOC_070302, CHD6, LLGL1, NEURL1B, KLHL38 and ACTR6 have been identified as modulators in adipogenic differentiation, which provide a valuable resource for further research of chicken lncRNAs and facilitate a better understanding of preadipocyte differentiation in the chicken (Gallus gallus) (78).

Until recently, most studies on EXs-lncRNAs throughout the world have focused on diagnostic markers of cancer and related diseases. However, there are relatively fewer studies on EXs-lncRNAs on adipogenic differentiation of adipocytes. A previous study in pancreatic cancer patients has shown that the uptake of plasma EXs-lncRNA-ROR by adipocytes can significantly inhibit its adipogenic differentiation through reducing the expression of mature adipocytes markers genes (79). Another study on patients with colorectal cancer has demonstrated that plasma EXs-lncRNA-HOTAIR can be actively taken up by adipocytes, which can further promote DKK1 expression by combining miR-218 to induce the adipogenic differentiation (80). These above results indicate that lncRNAs or EXs-lncRNAs of other tissues and organs can be involved in regulating physiological homeostasis and pathological processes of adipose tissue in health and diseases (**Table 3**).

**Table 3:** EXs and/or lncRNAs regulate adipogenic differentiation.

EXs and/or ncRNA	Donor cells/organs	Recipient cells/organs	Functions	Mechanisms	References
LncRNA-ADI	–	ADSCs	Promotes adipogenic differentiation	Inhibits miR-449a expression, increases the CDK6 translation and activates pRb-E2F1 pathway	(75)
LncRNA HOXA11-AS1	–	hADSCs	Reduces adipogenic differentiation ability and lipid accumulation	Knockdown of lncRNA HOXA11-AS1 inhibits C/EBP-α, DGAT2, CIDEC and perilipin	(76)
LncRNA LOXL1-AS1	–	hBMSCs	Switches from adipogenic differentiation to osteogenic differentiation	Promotes the expression of Hmga2 by inhibiting miR-196a-5p expression	(77)
EXs-lncRNA-ROR	Pancreatic cancer patients	Adipocytes	Inhibits its adipogenic differentiation	Reduces the expression of mature adipocytes markers genes	(79)
EXs-lncRNA-HOTAIR	Plasma of colorectal cancer	Adipocytes	Induces the adipogenic differentiation	Promotes DKK1 expression by combining miR-218	(80)

## Adipose tissue-derived EXs-ncRNAs and IR

Obesity is one of the leading causes of global morbidity and mortality of IR, T2DM, high blood pressure, high cholesterol, known as metabolic syndrome. It has been demonstrated in rodents and humans that obesity and IR are strongly associated with each other in the development of obesity-induced IR. At present, a large number of studies have shown that active substances in the body play an important role in the normal transfer of insulin signals between different metabolic organs. For example, in pancreatic beta cells, the miR-29 family member (miR-29s) can be secreted responsible to the high-level of free fatty acid (FFA). These β cell-derived EXs-miR-29s regulate glucose homeostasis by controlling hepatic glucose output, thereby inhibiting insulin signaling in the liver. Furthermore, blocking miR-29s expression in islet β cells can significantly reverse HFD-induced IR (81). In addition, the serum EXs of T2DM patients are rich in miR-20b-5p, which can lead to IR by reducing glycogen accumulation in skeletal muscle cells (82). EXs miRNAs from adipose tissue macrophage (ATM) of obese mice can increase FFA level in blood, impairing insulin sensitivity and enhancing IR (56). *In vivo* study of mice has also shown that the tail-vein injection of EXs-miR-29s secreted by islet β cells can lead to impaired hepatic insulin sensitivity (81).

Compared with wild-type mice, miR-223 deficient mice showed increased inflammatory response in adipose tissue and much more severe systemic IR under HFD (83), suggesting that miR-223 plays a key role in controlling adipose tissue inflammation and systemic IR. The expression level of miR-222 was significantly increased in serum EXs of patients with IR and T2DM, and the overexpressed EXs-miR-222 in mouse adipose tissue can significantly enhance IR in liver and skeletal muscle of HFD-induced obese mice by inhibiting IRS1 expression (84). These results suggest that EXs-miRNAs are closely related to obesity-related IR. In addition, adipocytes derived EXs-miR-27a can significantly induce IR in skeletal muscle by inhibiting the expression of PPAR-γ (85), suggesting that adipocytes derived EXs-miRNAs can regulate IR in other related tissues. Furthermore, although adipose tissue-derived EXs-ncRNAs play important roles in regulating obesity and obesity-induced IR, it is undoubted that changes in the internal microenvironment of adipose tissue in the pathological environment may have special effects on it and the secreted EXs (including the quantity or components). For example, it has been reported that adipose tissue dysfunction, like that seen in the obese and stress state, directly contributes to system-wide pathological metabolism by increasing the secretion of circulating EXs (86, 87).

It should be noted that EXs secreted by adipocytes mostly promote obesity-related IR, while insulin resistance can further promote adipogenic differentiation of adipocytes, and the level of EXs secretion increases significantly during adipocyte differentiation and maturation (88), which further promotes IR, thus generating a vicious cycle. However, liver secreted EXs-miR-130a-3p can improve glucose intolerance by inhibiting the expression of PHLPP2 in adipocytes, while specific knockout of miR-130a-3p is able to significantly increase the glucose level of HFD obese mice, and decreases the glucose tolerance and insulin sensitivity (55). These results indicate that hepatic EXs-miRNAs can significantly improve IR. In addition, compared with healthy individuals, the expression of miR-27a and miR-320 in plasma EXs of T2DM patients is significantly increased, suggesting that it may become possible to use EXs containing specific bioactive substances as serum diagnostic biomarkers to screen T2DM (89, 90).

ADEXs, as endocrine factors, can affect the metabolism and function of corresponding target organs by delivering bioactive molecules. Results showed that ADEXs-miRNAs can regulate the expression of PPAR-γ and FGF-21, and participate in the regulation of glucose tolerance and insulin sensitivity in the liver. Furthermore, in obese mice, ADEXs treatment is able to facilitate their metabolic homeostasis, improving insulin sensitivity by 27.8%, reducing obesity and alleviating hepatic steatosis (91). In skeletal muscle, ADEXs-miRNAs can improve insulin sensitivity and lipid oxidation ability through PPAR-γ and PGC1α, respectively. In the pancreas, ADEXs-miRNAs play a critical role in regulating islet β cell quality and insulin secretion. In the brain, ADEXs-lncRNA metastasis-associated lung adenocarcinoma transcription-1 (ADEXs-lncRNA MALAT1) regulates appetite and body weight by modulating mTOR signaling in hypothalamic POMC neurons. These above results demonstrate that adipose tissue-derived EXs and/or ncRNAs play important roles in the development of IR and metabolic diseases (**Table 4**).

**Table 4:** Adipose tissue-derived EXs and/or ncRNAs and IR.

EXs and/or ncRNA	Donor cells/organs	Recipient cells/organs	Functions	Mechanisms	References
EXs-miR-29s	Pancreatic beta cells	Liver	Regulates glucose homeostasis, impaires hepatic insulin sensitivity	Controls hepatic glucose output, inhibits insulin signaling	(81)
EXs-miR-20b-5p	The serum EXs of T2DM patients	Skeletal muscle cells	Induces IR	Reduces glycogen accumulation	(82)
EXs-miRNAs	Adipose tissue macrophage of obese mice	Mice	Enhances IR and increase FFA level in blood, impairs insulin sensitivity	Paracrine or endocrine regulation with robust effects on cellular insulin action, *in vivo* insulin sensitivity and overall glucose homeostasis	(56)
EXs-miR-222	Adipose tissue	Mice	Enhances IR in liver and skeletal muscle of HFD-induced obese mice	Inhibits IRS1 expression	(84)
EXs-miR-27a	Adipocytes	Skeletal muscle	Induces IR	Inhibits the expression of PPAR-γ	(85)
EXs-miR-130a-3p	Liver	Adipocytes	Improves glucose intolerance	Inhibits the expression of PHLPP2	(55)
EXs-miR-27a and miR-320	Plasma of T2DM patients	–	Serum diagnostic biomarkers to screen T2DM	–	(89, 90)
ADEXs	Adipose tissue	Liver	Participates in the regulation of glucose tolerance and insulin sensitivity in the liver.	Regulates the expression of PPAR-γ and FGF-21	(91)

## ADEXs-ncRNAs and the development of obesity and various cancers

ADEXs can not only regulate the functions of other related tissues and organs, but also affect adipose tissue itself through autocrine pathways. Previous studies have shown that ADSCs treated with ADEXs appears to have increased expression of key adipogenic genes PPAR-γ, aP2 and adiponectin, as well as higher intracellular lipid droplet accumulation (92). In addition, adipocyte EXs can promote the adipogenic differentiation of mouse stromal preadipocytes OP9 through TRPML1 (93). In addition, treatment with ADEXs can effectively activate the hedgehog (Hh) signaling pathway, especially during the HFD exposure, and inhibits both adipogenic differentiation and lipogenesis in ADSCs (94).

During the differentiation of mouse 3T3-L1 preadipocytes, the level of olic acid in EXs of cell supernatant was significantly increased, and the expression of FABP4, Pref-1 and adiponectin in EXs was significantly increased, suggesting that substances in adipocytes can be released into the extracellular medium through EXs (88). Furthermore, other studies have also shown that adipocytes can transfer lipids into surrounding macrophages through EXs, and increase the lipid accumulation in macrophage (95). The results reveal that lipid transfer can be carried out through EXs, providing new references for regulating the body fat and the lipid accumulation in adipocytes.

ADEXs play a regulatory role in many related organs and cells. In mice, adipocyte specific knockdown of Dicer, a key enzyme for miRNA biosynthesis, the content of miRNAs was significantly reduced in circulating EXs, suggesting that ADEXs accounted for a large proportion of circulating EXs (53). ADSCs EXs can improve T1DM by regulating immune cell response (96), alleviating podocyte injury (97) and promoting angiogenesis (98).

Cancer-associated adipocytes (CAAs), as a main component of the tumor-adipose microenvironment (TAME), have various functions, including remodeling the extracellular matrix and interacting with tumor cells or infiltrated leukocytes through a variety of mutual signals, Adipose tissue remodeling plays an important role in promoting tumors development, but the specific mechanisms are largely unknown. It has been shown that adipocyte EXs-miR-23a/b can regulate tumor growth by targeting the VHL/HIF axis, thus promoting the metastasis and growth of hepatocellular carcinoma cells (99). In addition, adipocyte EXs-circRNAs, especially circ_DB, can promote the growth of hepatocellular carcinoma by targeting USP7 deubiquitination (100), suggesting that adipocyte EXs-ncRNAs play a specific role in the development of tumor cells. Studies have also confirmed that adipocytes can exchange lipids into macrophages through EXs (95), providing energy for ovarian cancer cells (101), which suggest that adipocytes can transfer lipids outward through EXs, reduce lipid deposition and promote the development of ovarian cancer. Some other study has also shown that adipocyte EXs can significantly increase the drug resistance of cancer cells (102), indicating that the development of cancer can be inhibited by inhibiting adipocyte EXs production and/or blocking the uptake of adipocyte EXs by cancer cells. These above results show that EXs and EXs-ncRNAs can alter a wide range of cellular responses in recipient cells and play important pathophysiological roles in human cancers (103). In summary, ADEXs-ncRNAs participate in regulation of obesity and various cancers, which can be considered as crucial mediators of cancers, especially obesity-induced cancers (**Table 5**).

**Table 5:** ADEXs-ncRNAs and the development of obesity and various cancers.

EXs and/or ncRNA	Donor cells/organs	Recipient cells/organs	Functions	Mechanisms	References
EXs	Adipose tissue	ADSCs	Induces intracellular lipid droplet accumulation	Increases expression of key adipogenic genes PPAR-γ, aP2 and adiponectin	(92)
	Adipocytes	Mouse stromal preadipocytes OP9	Promotes the adipogenic differentiation	TRPML1-mediated lysosomal exocytosis	(93)
ADEXs	ADSCs	ADSCs	Inhibits adipogenic differentiation and lipogenesis	Activates the Hh signaling pathway	(94)
EXs	Adipocytes	Macrophage	Increases lipid accumulation	Transfers lipids into surrounding macrophages through EXs	(95)
EXs-miR-23a/b	Adipocytes	Hepatocellular carcinoma cells	Regulates tumor growth	Targets the VHL/HIF axis	(99)
Circ_DB			Promotes growth	Targets USP7 deubiquitination	(100)

## Conclusions

As a new research hotspot, EXs play important roles in the diagnosis and treatment of many diseases due to their extensiveness in the body and obtaining convenience. On one hand, EXs can be used as biomarkers for the diagnosis of various diseases, on the other hand, it can also be served as a treatment method, which is very likely to be applied as a natural carrier of drugs for clinical treatment in the future. However, in the actual application process, it is still difficult to label endogenous EXs, trace their origin and movement as well as identify specific targets, which makes the exploration of EXs function *in vivo* very complicated and needs to be further studied. This review summarized the mutual regulation and potential mechanisms of the EXs-ncRNAs between adipose tissue and other related tissues in obesity and metabolic diseases, hoping to elucidate the etiology of obesity and related metabolic diseases, and providing some references for the screening of new therapeutic targets.

## Author contributions

XX, XC and FL collected literatures. YC and JY wrote the manuscript and prepared the tables. HX and SP wrote the outline and critically revised the manuscript. All authors have read and given approval of the final manuscript.

## Funding

The study was supported by the National Natural Science Foundation of China (No. 32072809, 31501923), the Natural Science Foundation of Jiangsu Province (BK20211119, BK20150443), China Postdoctoral Science Foundation Funded Project (No.2015M581872), Postdoctoral Science Foundation Funded Project of Jiangsu Province (No.1501073A), the Top-level Talents Support Program of Yangzhou University (2018) (No.137080146), Postgraduate Research & Practice Innovation Program of Jiangsu Province (KYCX21_3275, KYCX22_3549), the Science and Technology Innovation Cultivation Fund of Yangzhou University (2019CXJ140), and the Priority Academic Program Development of Jiangsu Higher Education Institutions (PAPD).

## Acknowledgments

We thank Prof. Min Du of Washington State University for critical reading of the manuscript.

## Conflict of interest

The authors declare that the research was conducted in the absence of any commercial or financial relationships that could be construed as a potential conflict of interest.

## Publisher’s note

All claims expressed in this article are solely those of the authors and do not necessarily represent those of their affiliated organizations, or those of the publisher, the editors and the reviewers. Any product that may be evaluated in this article, or claim that may be made by its manufacturer, is not guaranteed or endorsed by the publisher.

## Abbreviations

ACTR6, actin related protein 6; ADSCs, adipose-derived stem cells; AP2, adipocyte protein 2; ATM, adipose tissue macrophage; BMSCs, bone marrow mesenchymal stem cells; CAAs, cancer-associated adipocytes; CAC, cancer-associated cachexia; CDK6, cyclin dependent kinase 6; C/EBP-α, CCAAT/enhancer binding protein alpha; C/EBP-β, CCAAT enhancer binding protein beta; CHD6, chromodomain helicase DNA binding protein 6; CIDEC, cell death-inducing DFFA-like effector C; circRNA, circular RNA; CML, chronic myeloid leukemia; DGAT2, acyl CoA: diacylgycerol acyltransferase 2; DKK1, dickkopf WNT signaling pathway inhibitor 1; ERK, extracellular regulated protein kinases; EXs, exosomes; EXs-AT, adipose tissue-derived exosomes; FABP4, fatty acid binding protein 4; FASN, fatty acid synthase; FFA, free fatty acid; FGF-21, fibroblast growth factor-21; hAMSCs, human adipose mesenchymal stem cells; hBMSCs, human bone marrow-derived mesenchymal stem cells; HepG2, human hepatocellular carcinomas; HFD, high fat diet; Hh, hedgehog; ILVs, intraluminal vesicles; IR, insulin resistance; IRS1, insulin receptor substrate 1; GC, gastric cancer; GLUT4, glucose transporter type 4; Klf4, kruppel-like factor 4; LncRNA, long non-coding RNA; LPL, lipoprotein lipase; MAP3K8, mitogen-activated protein kinase kinase kinase 8; mRNA, messenger RNA; mTOR, mammalian target of rapamycin; MVBs, multivesicular bodies; PGC1α, peroxisome proliferator-activated receptor gamma coactivator-1 alpha; PPAR-γ, peroxisome proliferator-activated receptor gamma; PRDM16, PR domain-containing 16; Pref-1, preadipocyte factor 1; SREBF1, sterol regulatory element binding transcription factor 1; SREBP1, sterol-regulatory element binding protein 1; T2DM, type 2 diabetes; TG, triglycerides; TGF-β, transforming growth factor-β; UCP1, uncoupling protein 1; USP7, ubiquitin specific peptidase 7; VDR, vitamin D receptor; VHL, von hippel-lindau; WISP2, Wnt-1-induced signaling protein-2.

## Glossary

**Table:**

ACTR6	actin related protein 6
ADSCs	adipose-derived stem cells
AP2	adipocyte protein 2
ATM	adipose tissue macrophage
BMSCs	bone marrow mesenchymal stem cells
CAAs	cancer-associated adipocytes
CAC	cancer-associated cachexia
CDK6	cyclin dependent kinase 6
C/EBP-α	CCAAT/enhancer binding protein alpha
C/EBP-β	CCAAT enhancer binding protein beta
CHD6	chromodomain helicase DNA binding protein 6
CIDEC	cell death-inducing DFFA-like effector C
circRNA	circular RNA
CML	chronic myeloid leukemia
DGAT2	acyl CoA: diacylgycerol acyltransferase 2
DKK1	dickkopf WNT signaling pathway inhibitor 1
ERK	extracellular regulated protein kinases
EXs	exosomes
EXs-AT	adipose tissue-derived exosomes
FABP4	fatty acid binding protein 4
FASN	fatty acid synthase
FFA	free fatty acid
FGF-21	fibroblast growth factor-21
hAMSCs	human adipose mesenchymal stem cells
hBMSCs	human bone marrow-derived mesenchymal stem cells
HepG2	human hepatocellular carcinomas
HFD	high fat diet
Hh	hedgehog
ILVs	intraluminal vesicles
IR	insulin resistance
IRS1	insulin receptor substrate 1
GC	gastric cancer
GLUT4	glucose transporter type 4
Klf4	kruppel-like factor 4
LncRNA	long non-coding RNA
LPL	lipoprotein lipase
MAP3K8	mitogen-activated protein kinase kinase kinase 8
mRNA	messenger RNA
mTOR	mammalian target of rapamycin
MVBs	multivesicular bodies
PGC1α	peroxisome proliferator-activated receptor gamma coactivator-1 alpha
PPAR-γ	peroxisome proliferator-activated receptor gamma
PRDM16	PR domain-containing 16
Pref-1	preadipocyte factor 1
SREBF1	sterol regulatory element binding transcription factor 1
SREBP1	sterol-regulatory element binding protein 1
T2DM	type 2 diabetes
TG	triglycerides
TGF-β	transforming growth factor-β
UCP1	uncoupling protein 1
USP7	ubiquitin specific peptidase 7
VDR	vitamin D receptor
VHL	von hippel-lindau
WISP2	Wnt-1-induced signaling protein-2
